# Group Psychodynamic Counselling as a Clinical Training Device to Enhance Metacognitive Skills and Agency in Future Clinical Psychologists

**DOI:** 10.5964/ejop.v14i2.1528

**Published:** 2018-06-19

**Authors:** Cristiano Scandurra, Simona Picariello, Daniela Scafaro, Vincenzo Bochicchio, Paolo Valerio, Anna Lisa Amodeo

**Affiliations:** a Department of Neurosciences and Reproductive and Odontostomatological Sciences, University of Naples Federico II, Naples, Italy; b SInAPSi Center, University of Naples Federico II, Naples, Italy; c Department of Humanistic Studies, University of Calabria, Rende, Italy; d Department of Humanistic Studies, University of Naples Federico II, Naples, Italy; Department of Psychology, Webster University Geneva, Geneva, Switzerland; Psychology Department, College of New Rochelle, New Rochelle, USA

**Keywords:** psychodynamic counselling, group, metacognition, agency, clinical psychology

## Abstract

Metacognitive skills and agency are among the main psychological abilities a clinical psychologist should have. This study aimed to assess the efficacy of group psychodynamic counselling as a clinical training device able to enhance metacognitive skills and agency in final-year undergraduates in clinical psychology within an educational context. Thirty-three final-year students of clinical psychology participated in an experiential laboratory lasting two months. Participants completed measures regarding metacognitive skills and agency at pre-, post-treatment, and 3-month follow-up assessment. The results suggested that group psychodynamic counselling made students feel more capable of recognizing emotional states, understanding causal relationships, inferring mental states of others in terms of beliefs, desires, intentions, and expectations, and thinking critically. Furthermore, the group psychodynamic counselling helped students to feel more able to derive pathways to desired goals and to motivate themselves via agency thinking to use those pathways. Thus, the study confirmed the efficacy of group psychodynamic counselling as a clinical training device able to enhance metacognitive skills and agency in future clinical psychologists.

Undergraduates in clinical psychology must acquire specific clinical competencies, such as conduction of clinical interviews, taking charge of psychological distress, health promotion in a preventive perspective, and so on. Learning these psychological-clinical skills requires that students deeply reflect on the role they will take in different health care contexts. These reflective skills must be supported and encouraged during university training, in which students may project themselves into future work scenarios (Freda, Esposito, & Quaranta, 2015).

Among others, two fundamental psychological skills a clinical psychologist should improve are metacognitive skills and agency (Sheikh, Milne, & MacGregor, 2007). As we will discuss below, these skills include reflective ability, that is, the capacity to critically analyze experience, a fundamental skill for future clinical psychologists (Freda & Esposito, 2017). With the aim of promoting these skills in a group of future clinical psychologists, we conducted a learning laboratory with undergraduates at the beginning of their last year of the Master’s Degree in Clinical Psychology program at the University of Naples Federico II (Italy), which is strongly characterized by a psychodynamic foundation. Due to this specific foundation, we decided to use the group psychodynamic counselling as a clinical training device, assessing its effectiveness in enhancing metacognitive skills and agency.

Within clinical settings, it is common to find studies on cognitive-behavioral interventions aimed at enhancing patients’ metacognitive skills (e.g., Lopez, Floyd, Ulven, & Snyder, 2000). Recently, studies assessing the effectiveness of psychodynamic interventions in enhancing these skills in clinical settings have also been conducted (e.g., Harder & Folke, 2012; Rosenbaum et al., 2012). Within educational contexts, however, although some research has demonstrated the efficacy of learning cognitively-oriented laboratories in enhancing metacognitive skills and agency in students (e.g., Kuiper, 2002), to our knowledge no previous studies have assessed whether a psychodynamically-oriented laboratory can effectively enhance these skills in future clinical psychologists. Thus, the innovation of the current study is in its assessment of the effectiveness of a psychodynamic intervention carried out within an educational context to enhance metacognitive skills and agency in a non-clinical sample (i.e., students in clinical psychology).

In the following paragraphs, we report theoretical definitions of metacognition and agency, highlighting their fundamental role for clinical psychologists and their relationship with reflective ability. Subsequently, we report evidence for the importance of conducting learning laboratories aimed at enhancing metacognition and agency, and then we describe the theory and technique for using group psychodynamic counselling with undergraduates, both in clinical and educational contexts.

## Metacognition and Agency: Their Role for Clinical Psychologists and Relationship With Reflective Ability

Metacognitive skills are conceptualized as an individual’s knowledge and regulation over one’s own cognitions (Flavell, 1979) and they lead to monitor progress through goals, to identify strengths and weaknesses, and to modify learning strategies in agreement with more favorable outcomes (Ford, Smith, Weissbein, Gully, & Salas, 1998; Quattropani, Lenzo, Mucciardi, & Toffle, 2015). More specifically, Semerari et al. (2008) defined metacognition as a set of abilities that allows to: (a) Attribute and recognize mental states to oneself and to the others; (b) Reflect upon and reason with mental states; (c) Use information about mental states to decide upon solutions for psychological and interpersonal problems and cope with personal suffering. According to this definition, it is reasonable to believe that dysfunctional metacognitive beliefs are associated with the development and maintenance of psychological disorders, such as anxiety disorder, social phobia, panic disorder, anorexia nervosa, and so on (e.g., Quattropani, Lenzo, & Filastro, 2017; Quattropani, Lenzo, Mucciardi, & Toffle, 2016; Wells & Carter, 2001).

On the basis of the definition by Semerari et al. (2008), adequate metacognitive ability is shown through the perception of oneself as an intentional agent, through the ability to reflect and reason on one’s own and others’ mental states, and through the ability to use psychological knowledge to regulate action plans and manage psychological problems (Alaimo & Schimmenti, 2013). As metacognitive skills help to inform decisions and actions, and lead to a monitoring phase (Sheikh et al., 2007), we believe that they are fundamental for clinical psychologists who are faced with complex circumstances in which they should take charge of pathological situations, try to promote health in diverse contexts, and constantly rethinking about their actions and decisions.

Metacognition is somehow connected with agency. Indeed, agency represents an on-going process through which one’s own actions are monitored and might be self-corrected ensuring that they are goal directed (Paternoster & Pogarsky, 2009). As stated by Metcalfe and Greene (2007), “the profound implications of agency are predicated on the idea that people are […] able to monitor their own agency, that is, to make metacognitive assessments about when and whether they are, themselves, in control” (p. 184). Thus, as suggested again by Sheikh et al. (2007), agency is a fundamental person-centered capacity for the active engagement of clinical psychologists in clinical processes and decisions. This is true also for students, whose *agentic engagement* (Reeve & Tseng, 2011) in learning activities represents a fundamental learning process (Christenson, Reschly, & Wylie, 2011), predicting better academic progress and achievements (Ladd & Dinella, 2009).

On the basis of the previous definitions, metacognitive skills and agency should be viewed as two dimensions including the reflective ability (Freda & Esposito, 2017). To this end, Sheikh et al. (2007) clearly stated that metacognition, that is, the capacity through which the mind thinks about itself (“thinking about thinking”), informing decisions and actions, is a reflective capacity which leads to a monitoring phase that might help to change the direction of one’s own decisions and actions. On the other hand, Paternoster and Pogarsky (2009) defined agency as “an intentional activity directed toward some goal [which involves] thoughtfully reflective behavior” (pp. 111-112), clearly linking agency to a reflective ability.

Development of reflective ability is crucial in clinical psychology students’ education and in their future work because it allows them to analyze and re-analyze their own activities (Dallos & Stedmon, 2009), and critically analyze experiences (Freda & Esposito, 2017). As a consequence, self-reflection is considered a mandatory and non-optional activity for an ethical and effective clinical psychology practice (Lavender, 2003). For this reason, it seems crucial to train future clinical psychologists in enhancing their reflective abilities.

## Learning Laboratories Enhancing Students’ Metacognition and Agency

Hofstein, Kipnis, and Kind (2008) highlighted the importance of promoting metacognitive skills and agency within learning laboratories. Indeed, once learned, metacognition supports lifelong reflective thinking in divergent situations, helps in the management of ambiguity and problems, promotes a sense of responsibility for one’s own actions, and increases a sense of self-confidence for rapid decision-making (Kuiper, 2002).

Much research in the area of science education has shown that metacognitive processes are able to promote meaningful learning so that students improve their ability to apply their learning in a new context (e.g., Rickey & Stacey, 2000; Thomas & McRobbie, 2001), thus becoming an active agent. Most of these researchers agree that metacognitive processes are able to promote the problem-solving processes and the students’ awareness of their cognitions. For example, Kuiper (2002) described an eight-week training addressed to new graduate nurses who were transiting into a workplace dimension. Self-regulated learning strategies proved useful in enhancing metacognitive critical thinking abilities and helping nursing graduates improve their thinking strategies. Again, Abd-El-Khalick and Akerson (2009) described the influence of a training in metacognitive strategies on the development of pre-service elementary teachers’ views of nature of science. The authors reported that those participating in this training developed a more informed view of the target aspects of nature of science. To our knowledge, no previous studies dealt with laboratories enhancing metacognitive skills and agency addressed to final-year undergraduates in clinical psychology programs from a psychodynamic point of view. Similar to the works of Kuiper (2002) and Abd-El-Khalick and Akerson (2009), participants taking part in our training laboratory were also transiting into a workplace dimension. Furthermore, the particular attention the psychodynamic approach gives to emotions is in line with the most recent theories about metacognition, considered not only as thinking about thinking, but also as thinking about emotion (e.g., Eichbaum, 2014).

Promoting these skills in final-year undergraduates can therefore mean increasing their reflective ability on their own mental and emotional states connected to becoming clinical psychologists, helping them to project themselves toward future work scenarios. To this end, Freda et al. (2015) argued that students’ learning of clinical skills should go through a reflection process, that is, the psychological process focusing on deeper recognition of oneself as having an active role in relationships, giving them a subjective meaning. Furthermore, Freda et al. (2015) stated that this is not just a cognitive process, but implies that students come into contact with their and others’ emotions, assuming a reflective position, or rather, understanding that individuals’ behaviors are a consequence of intentional mental states.

Within this context, the use of group formats has been considered the best approach to promote reflection processes (Amodeo, Picariello, Valerio, & Scandurra, 2018; Freda et al., 2015) and, thus, to enhance metacognitive skills and agency. Group is a device that facilitates this process because participants become bearers of different representations of the same experience—in this case, of being final-year undergraduates in clinical psychology—and the individual has to deal with different points of view, putting in motion a reflective and self-observational process that can bring change to a rigid position (Amodeo, Picariello, Valerio, Bochicchio, & Scandurra, 2017). Indeed, groups have the potential to promote fruitful exchange between participants, thanks to the mirroring processes that groups activate (e.g. Bion, 1961) and the role of counsellor as *creative-social-mirror* (Fonagy & Target, 1997) who bears in mind the mental states of the others, translating them into a comprehensive language (Esposito, Ribeiro, Alves, Gonçalves, & Freda, 2017a; Esposito, Ribeiro, Gonçalves, & Freda, 2017b). Mirroring processes have to be intended as those processes occurring in groups that allow individuals to intersubjectively reflect on one’s own thoughts and feelings “by recognizing their experience in others as they empathize with the other and respond internally as if that person were the self” (Esposito et al., 2017b, p. 392). Thus, as argued by Freda et al. (2015), the group represents a dimension in which reflective processes take place very intensively. Indeed, relationships between group members and between participants and facilitator become an object for reflection and a chance to reflect on those interactions occurring in the *here and now* of the group setting. We believe that whether these processes become an object of reflection, an increase in metacognitive skills and agency might be produced. This is the reason why we decided to use a group device to increase these abilities.

## Group Psychodynamic Counselling as a Clinical Training Device

Group psychodynamic counselling is a short-term clinical intervention delivered in a group format through a limited number of sessions (from four to six). It is aimed at providing clients with opportunities to understand an actual conflictual situation more clearly, helping them to cope with it and to make choices and changes accordingly (Amodeo, Liccardo, Tortono, & Valerio, 2008). In this work we make reference to the theoretical model used by psychotherapists working at the Tavistock Clinic in London (e.g., Copley, 1976; Salzberger Wittenberg, 1986), whose roots are in Bion’s work within institutions (e.g., Bion, 1961). This is the reason why we use the term “counselling” and not “counseling.”

Most scientific work in this field refers to the individual psychodynamic counselling, an intervention considered particularly suitable for young adults because, for its shortness, it does not offer incentives for regression and dependence in a developmental period during which individuals are trying to act out a separation process from parental internal objects (Adamo, Sarno, Preti, Fontana, & Prunas, 2010; Hetherington, 1999; Noonan, 1983; Scandurra, 2016). The suitability of psychodynamic counselling for young adults led researchers and counsellors to consider this intervention particularly useful for university students (e.g. Adamo, 1999; Copley, 1976; Richards, 1999), due to their specific developmental stage during which they are constructing a professional identity and the job market is around the corner.

Group psychodynamic counselling differs from the individual counselling mainly because of the use of the group as a work tool. Within this group format, a silent observer acting as a catalyst for persecutory and superego anxieties and a container for the group’s frustrations (Chiodi, Di Fratta, & Valerio, 2009) supports the counsellor who facilitates the intervention. Amodeo et al. (2017) reported a learning laboratory experience where they used group psychodynamic counselling as a clinical training methodology to reinforce well-being and academic identity in a group of final-year undergraduates in clinical psychology, demonstrating that this intervention can be productively used beyond a clinical context within university and educational settings with students who are becoming clinical psychologists to provide them with new tools and skills.

During the first counselling session, the “work couple”—the counsellor and the observer—were introduced by the counsellor and the participants were told:

This is a space in which we have the possibility to think and reflect about your status as final-year undergraduates in clinical psychology. In this space it is possible to say anything you think, using fantasies, thoughts, and dreams. Whatever emerges in this space will belong to the group and thus be treated as group material.

Thus, the counsellor provides a specific working focus (students are invited to think about their status of final-year undergraduates in clinical psychology) and at the same time open working focus (they can think of this in all ways they feel as appropriate for that specific group) leading participants to reflect together and to incentivize a deep exploration on their actual identity status. The counsellor uses classical psychodynamics tools like interpretations and clarifications, paying particular attention to transference and counter-transference phenomena. Notwithstanding that, differently from a long-lasting group psychodynamic intervention, the counsellor is more active, facilitating narrations, memories, and debates between participants. Furthermore, in the classic psychodynamic counselling, no working focus is used, allowing clients to be totally free to say what they want and need.

Within a Bionian framework (Bion, 1961), the group is considered as a whole and particular attention is given to the interpretations of its conscious and unconscious mental and emotional states. The Bionian approach has its roots within Freudian psychoanalysis, even though its contribution on the psychoanalytic theory of schizophrenia and group phenomena laid the foundations of a somehow divergent current. Within this framework, every group is seen as operating in two contrasting and co-existing ways. The “work group” is the group mental functioning which deals with the primary task, that is, what the group is called upon to accomplish; this mental state implies contact with reality, the tolerance of frustration, and the control of the drives. The “basic assumptions,” on the other hand, are intense and primitive emotions that dominate the group’s unconscious functioning and determine the ways in which the group tries to reach a common goal. In other words, the “basic assumptions” are omnipotent and magical group fantasies that are often completely contrary to the conscious opinions of the members. Within such a complex frame, the counsellor identifies major themes (both conscious and unconscious) and gives them back to the group, starting from the *here and now* to the *there and then*. Differently, the observer assumes the function of a receptacle for group projections, making group anxieties more manageable and facilitating the counsellor’s work. This complex mechanism allows the group to project its persecutory and superego function on the observer, relieving resistances and protecting the counsellor from aggressive fantasies, so that counsellor interventions might be perceived as good nourishment. This function is essential due to the short duration of the intervention. Furthermore, the observer has the task of writing observational reports at the end of each session, and these narrative materials become the reflection materials of a supervision where all staff members discuss about the psychodynamic process with an external psychodynamic counsellor. In this way, the supervisor may fulfill the function of *rêverie* (Bion, 1962).

## The Current Study

The aim of this study was to assess the efficacy of group psychodynamic counselling in enhancing perceived metacognitive skills and agency in final-year undergraduates in clinical psychology. Previous studies have already demonstrated that both cognitive-behavioral treatments (e.g., Lopez et al., 2000) and psychodynamic clinical interventions (Harder & Folke, 2012; Rosenbaum et al., 2012) were effective in enhancing metacognitive skills and agency in clinical samples. The same was found in the case of learning cognitively-oriented laboratories, which were effective in enhancing metacognitive skills and agency in non-clinical sample, such as students (Abd-El-Khalick & Akerson, 2009; Kuiper, 2002). Thus, informed by results achieved in these previous studies, we hypothesized that also a learning psychodynamically-oriented laboratory conducted through a psychodynamic device (i.e., group psychodynamic counselling) would be effective in enhancing metacognitive skills and agency in a non-clinical sample (i.e., students in clinical psychology). Specifically, we expected that the group psychodynamic counselling described above might enhance perceived metacognitive skills, as it introduces reflective processes on mental and emotional states (Amodeo et al., 2017), and these processes are treated in a group format that by its nature promotes a deep exploration of these states thanks to mirroring processes (Esposito et al., 2017b; Freda et al., 2015). Furthermore, we also expected that this process allows participants to perceive themselves as more intentional agents, or rather to view themselves as agents able to provoke mental and emotional states in others, regulating on this basis their action plans and managing better psychological problems and conflicts (Paternoster & Pogarsky, 2009; Sheikh et al., 2007). Indeed, participants took part in a laboratory of clinical psychology called “Learning from Experience,” based on the same Bionian (1962) theoretical and clinical concepts. According to Bion (1962), we can learn from experiences only if a transformation of the emotional experience occurs and if individuals living their experiences become able to rework it subjectively, actively integrating new learned concepts into their own identity in a continuous cycle of action, research, and reflection. Thus, beyond an unconscious work on these contents, it is necessary to promote a reflective thinking upon them.

## Method

### Participants and Procedures

Thirty-three students (9 males and 24 females) with an average age of 24.89 (*SD* = 3.73) took part in a university in the last year of a degree course in clinical psychology at the University of Naples Federico II.

The laboratory lasted two months and was organized into six 8-hour sessions. Due to the high number of participants, they were randomly divided into two “small groups”—one consisting of 16 and the other of 17 participants—and each group participated in six sessions of group psychodynamic counselling that took place at the beginning of classes and lasted 1 hour and 15 minutes. Therefore, the only difference between the two groups was the “work couple” (i.e., the counsellor and the observer) and, thus, the personal management style of the counsellor. Notwithstanding that, both counsellors (as well as both observers) were psychodynamic-oriented psychotherapists with a solid training in psychodynamic psychotherapy with children, adolescents, or adults, based in particular on the Tavistock model.

Each of the six days began with a psychodynamic group counselling session, with the exception of the last day, during which the session was conducted at the end of the day with the aim of carefully working through and holding separation anxieties. At the end of each group psychodynamic counselling session—with the exception of the last session where the following structure was inverted—the two groups were joined together in a long day of classes conducted by a teacher in clinical psychology who worked through traditional methods, such as readings related to psychodynamic counselling or clinical cases.

All data were collected in accordance with the Italian Law on Privacy and Data Protection 196/2003 and became property of the Department of Humanistic Studies of the University of Naples Federico II. They were stored in a database accessible only to the Principal Investigator. The study was designed to respect all principles of the Declaration of Helsinki on Ethical Principles for Medical Research Involving Human Subjects. In accordance with Italian law, informed consent was obtained before starting the intervention. Furthermore, all participants gave consent to report their narratives in a scientific manuscript.

### Measures

#### Metacognitive Functions Screening Scale (MFSS)

The MFSS is a 30-item measure assessing metacognitive functions (Alaimo & Schimmenti, 2013). Alaimo and Schimmenti (2013) defined metacognition as the set of abilities that allow individuals to: (1) Attribute and recognize mental states starting with facial expressions, somatic states, behaviors, and actions; (2) Reflect and think about mental states; (3) Use information on mental states to solve problems or psychological and interpersonal conflicts and to master the subjective suffering. Based on this definition, Alaimo and Schimmenti (2013) created a new measure constituted by four subscales: (1) CRE, the ability to recognize Emotional States and describe personal and social emotions (e.g., “I often do not know what adjective to use to describe my emotion”); (2) CRC, the ability to understand Causal Relationships to build relationships between behaviors and goals to reach (e.g., “I hate to think today about what may happen tomorrow”); (3) CDD, the ability to Judge the Distance of objects from one another and from ourselves, or rather the ability to infer the mental state of another person in terms of beliefs, desires, intentions, and expectations (e.g., “I often find myself unable to tune in with the emotions that people with whom I come into contact experience”); and (4) CDP, the ability to Ponder Situations and Problems, that is, the critical thinking and the ability to evaluate situations and problems to better apply previous experiences to actual events (e.g. “When I face with important or delicate situations, I always try to take advantage of previous experiences to avoid negative consequences”). In our sample the values of Cronbach’s alpha for each subscale at pre-, post-treatment, and 3-month follow-up assessment were as follows: CRE (α_pre_ = .77; α_post_ = .82; α_follow-up_ = .84), CRC (α_pre_ = .71; α_post_ = .68; α_follow-up_ = .75), CDD (α_pre_ = .68; α_post_ = .62; α_follow-up_ = .75), and CDP (α_pre_ = .64; α_post_ = .65; α_follow-up_ = .63). The response options ranged from “strongly disagree” to “strongly agree” on a 4-point Likert scale.

#### Adult Hope Scale (AHS)

The AHS is a 12-item measure of a respondent’s level of hope (Snyder et al., 1991; Italian adaption by Ferrari, Nota, & Soresi, 2010). Snyder et al. (1991) defined hope as the perceived capability to derive pathways to desired goals and motivate oneself via agency thinking to use those pathways. Higher hope has been found to be related to better outcomes in academics, athletics, physical health, psychological adjustment, and psychotherapy. This scale is divided into two subscales comprising Snyder’s cognitive model of hope: Agency, that is, the goal-directed energy (e.g., “I energetically pursue my goals”) and Pathway, or planning to accomplish goals (e.g., “I can think of many ways to get out of a jam”). In our sample the values of Cronbach’s alpha for each subscale at pre-, post-treatment, and 3-month follow-up assessment were as follows: Agency (α_pre_ = .69; α_post_ = .68; α_follow-up_ = .62) and Pathway (α_pre_ = .67; α_post_ = .80; α_follow-up_ = .81). The response options ranged from “definitely false” to “definitely true” on a 8-point Likert scale.

#### Statistical Analyses

All analyses were performed using SPSS 20. A one-way within-subjects (repeated measures) ANOVA was conducted to compare the effect of intervention on the dependent variables—metacognitive functions and adult hope (agency and pathways). Bonferroni adjustments were applied evaluating the subscale questionnaires. When the sphericity assumption was violated, Greenhouse Gressier statistics were used and reported. Cohen’s (1988) classification scheme for the effect size (η^2^; small effect = .01, medium effect = .06 and large effect = .14) was used to index and interpret the proportion of variance explained by the variables. Finally, to verify whether the management style of the two different counsellors could have affected the results, a one-way between-subjects ANOVA was performed, considering the group as the reference variable. Furthermore, with the aim of controlling for the potential effects of participants’ age (e.g., Justice & Dornan, 2001) and gender (e.g., Bidjerano, 2005)—especially considering the Italian context (Amodeo, Vitelli, Scandurra, Picariello, & Valerio, 2015; Scandurra, Amodeo, Bochicchio, Valerio, & Frost, 2017; Scandurra, Amodeo, Valerio, Bochicchio, & Frost, 2017; Scandurra, Bacchini, Esposito, Bochicchio, Valerio, & Amodeo, 2018; Scandurra, Braucci, Bochicchio, Valerio, & Amodeo, 2017; Scandurra, Mezza, Bochicchio, Valerio, & Amodeo, 2017; Scandurra, Picariello, Valerio, & Amodeo, 2017; Vitelli et al., 2017a; Vitelli et al., 2017b)—on metacognitive skills and agency, these variables were included as covariates in a one-way within-subjects ANOVA.

## Results

No difference based on management style was detected in the one-way between-subjects ANOVA, indicating that the management style of the two counsellors was not a significant variable to explain changes in outcome dimensions. Furthermore, neither age nor gender were significant as covariates, indicating that neither dimension significantly impacted the changes detected. Thus, we provide only results for the one-way within-subjects ANOVA. In Table 1, means, standard deviations, and within-subjects effects test for intervention outcome measures at pre-, post-treatment, and 3-month follow-up assessment are reported. Instead, in Table 2, pairwise comparisons with Bonferroni correction from pre- to post-intervention, post- to follow-up assessment, and pre- to follow-up assessment on the outcome measures are reported.

### Metacognitive Functions

**Figure 1:**
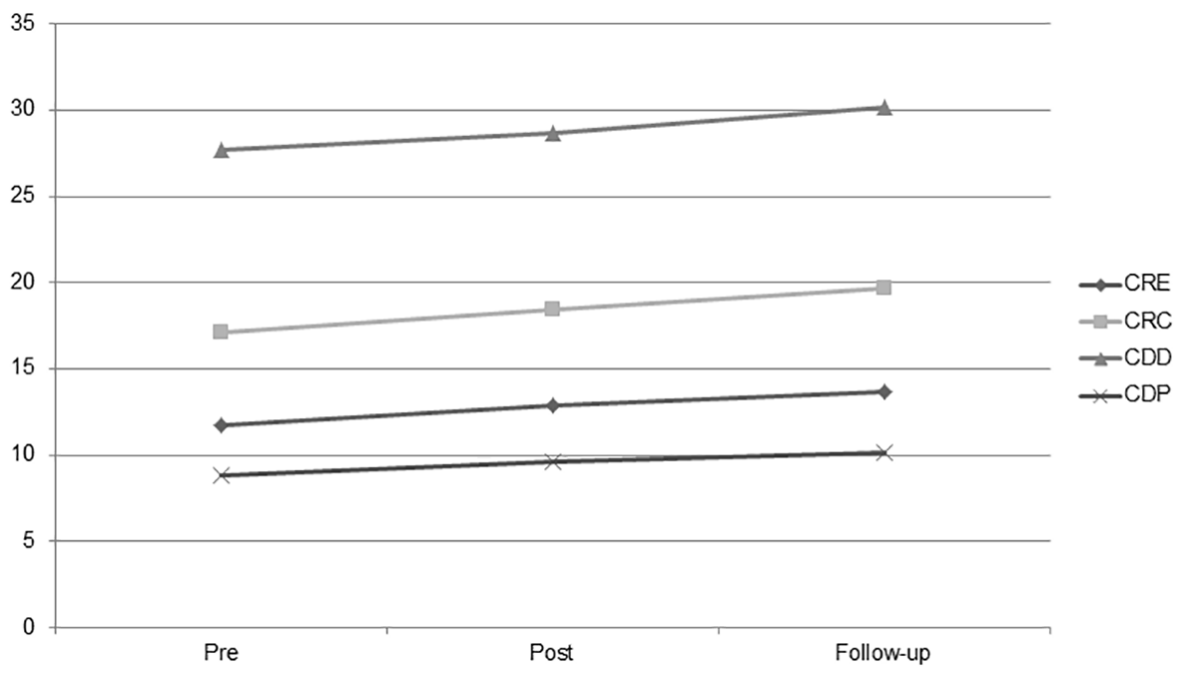
Changes of metacognitive functions at pre-, post-treatment, and 3-month follow-up assessment. *Note.* CRE = ability to recognize emotional states; CRC = ability to understand causal relationships; CDD = ability to judge the distance of objects; CDP = ability to ponder situations and problems.

For the MFSS measure, the repeated measures ANOVA revealed a significant effect for time for each subscale (see Table 1). Post hoc tests using the Bonferroni correction revealed significant time effects from pre- to post-, post- to follow-up, and pre- to follow-up assessment on all subscales. An increase in each time of the intervention was detected. This suggests that significant change in perceived metacognitive functions was not only achieved at post-assessment and maintained at follow-up, but additionally, improvement occurred from post- to follow-up assessment (see Table 2). Results are also reported in graphic form (see Figure 1).

### Hope

**Figure 2:**
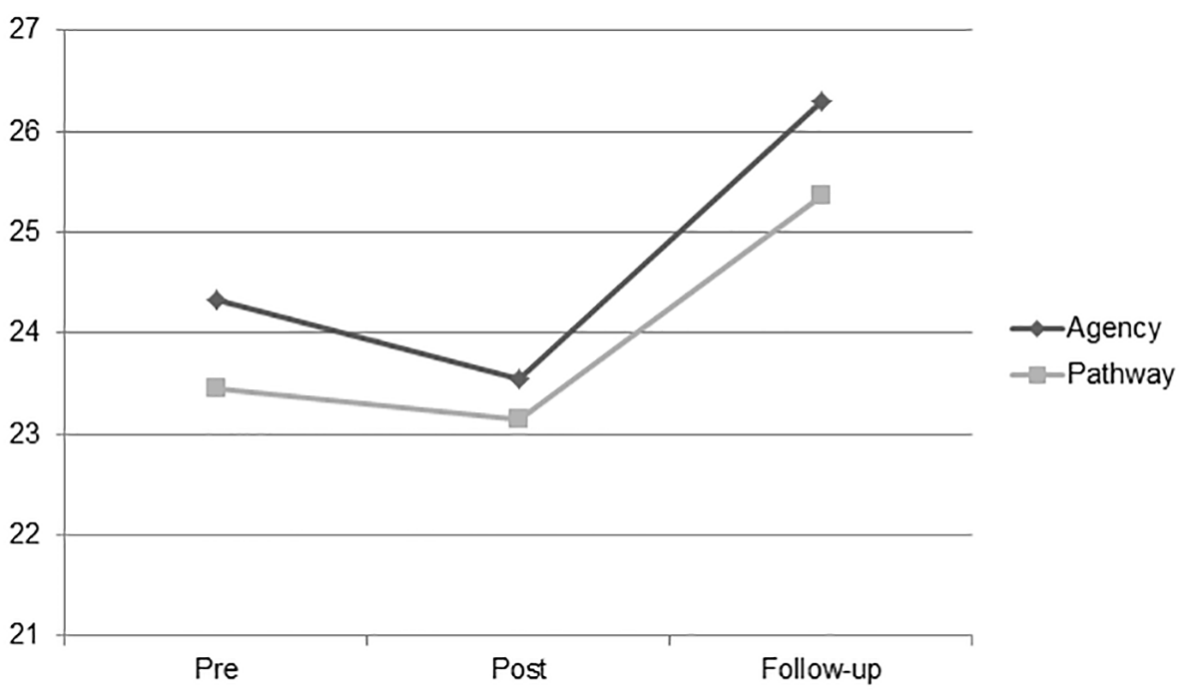
Changes of agency and pathways (Adult Hope Scale) at pre-, post-treatment, and 3-month follow-up assessment.

For the AHS measure, the repeated measures ANOVA revealed a significant effect for time for each subscale (see Table 1). Post hoc tests using the Bonferroni correction revealed significant time effects from post- to follow-up and pre- to follow-up assessment on both subscales, but none from pre- to post-intervention (see Table 2). The levels of both AHS subscales decreased from pre- to post-intervention and increased from post- to follow-up and from pre- to follow-up assessments. Thus, it seems that the group psychodynamic counselling caused a statistically significant increase in the goal-directed energy (Agency) and in the planning to accomplish goals (Pathways), but not immediately after the intervention, where these dimension levels decreased. Results are also reported in graphic form (see Figure 2).

**Table 1:** Means, Standard Deviations, and Within-Subjects Effects Test for Intervention Outcome Measures at Pre-, Post-Treatment, and 3-Month Follow-Up Assessment

Measure	Pre		Post		Follow-up		*F*	*df*	η^2^
	*M*	*SD*	*M*	*SD*	*M*	*SD*			
CRE	11.76	3.21	12.91	2.44	13.71	2.90	13.49***	1.51	.30
CRC	17.12	2.95	18.42	2.51	19.64	1.77	39.83***	1.56	.55
CDD	27.73	2.85	28.67	2.87	30.18	2.51	46.08***	1.36	.59
CDP	8.85	1.77	9.61	1.37	10.15	1.26	37.14***	1.41	.54
Agency	24.33	3.84	23.54	2.84	26.30	2.41	31.70***	1.68	.50
Pathway	23.45	3.53	23.15	3.33	25.36	2.83	21.80***	1.50	.40

*Note.* CRE = ability to recognize emotional states; CRC = ability to understand causal relationship; CDD = ability to judge the distance of objects; CDP = ability to ponder situations and problems.

*p < .05. **p < .01. ***p < .001.

**Table 2:** Pairwise Comparisons From Pre to Post, Post to Follow-Up, and Pre to Follow-Up Assessments on the Outcome Measures in Training Participants (N = 33)

Measure	*MD*	*SE*	*p*	95% CI
CRE				
Pre to post	1.15	0.29	.001	[0.41, 1.89]
Post to follow-up	0.79	0.35	.008	[-0.08, 1.67]
Pre to follow-up	1.95	0.47	.001	[0.76, 3.14]
CRC				
Pre to post	1.29	0.25	< .001	[0.66, 1.92]
Post to follow-up	1.22	0.23	< .001	[0.63, 1.81]
Pre to follow-up	2.51	0.35	< .001	[1.63, 3.39]
CDD				
Pre to post	0.94	0.14	< .001	[0.58, 1.30]
Post to follow-up	1.51	0.30	< .001	[0.75, 2.28]
Pre to follow-up	2.45	0.29	< .001	[1.71, 3.19]
CDP				
Pre to post	0.76	0.14	< .001	[0.39, 1.12]
Post to follow-up	0.54	0.11	< .001	[0.27, 0.81]
Pre to follow-up	1.29	0.19	< .001	[0.81, 1.78]
Agency				
Pre to post	-0.79	0.43	.221	[-1.86, 0.29]
Post to follow-up	2.76	0.30	< .001	[2.00, 3.52]
Pre to follow-up	1.97	0.33	< .001	[1.13, 2.81]
Pathway				
Pre to post	-0.30	0.24	.649	[-0.91, 0.30]
Post to follow-up	2.20	0.39	< .001	[1.22, 3.19]
Pre to follow-up	1.90	0.43	< .001	[0.82, 2.99]

*Note*. *MD* = Mean Difference; *SE* = Standard Error; CI = Confidence Interval; CRE = ability to recognize emotional states; CRC = ability to understand causal relationships; CDD = ability to judge the distance of objects; CDP = ability to ponder situations and problems.

## Discussion

The aim of this study was to assess the efficacy of group psychodynamic counselling in enhancing metacognitive skills and agency in final-year undergraduates in clinical psychology within an educational context. The results suggest that this kind of clinical training device is an effective method to achieve those objectives as the levels of these dimensions increased after the intervention and, above all, after the three-month follow-up assessment. Specifically, it seems that group psychodynamic counselling made students feel more capable of recognizing emotional states, understanding causal relationships, inferring mental states of others in terms of beliefs, desires, intentions, and expectations, and thinking critically, evaluating situations and problems to better apply the previous experiences with actual events. Furthermore, it also emerged that this clinical training device helped students to feel more able to derive pathways to desired goals and to motivate themselves via agency thinking to use those pathways.

The above mentioned psychological skills were higher after three-month follow-up than immediately after the treatment, indicating that group psychodynamic counselling activated psychological processes that seemed to lead to an ongoing positive change, continuing also after the end of the intervention. Although participants in the current study cannot be considered as a clinical sample, it is worth noting that this constant growth trend has been detected in meta-analytic studies assessing the effectiveness of long- and short-term psychodynamic psychotherapy (e.g., Abbass, Hancock, Henderson, & Kisely, 2014; de Maat, de Jonghe, Schoevers, & Dekker, 2009; Leichsenring & Rabung, 2008). Indeed, from these studies it is possible to note that benefits produced by psychodynamic interventions endured and increased with time, in contrast to the effects of other non-psychodynamic empirically supported psychotherapies where the benefits seemed to tend to decay with time for the most common disorders, such as depression and anxiety (Shedler, 2010). Notwithstanding these considerations, as previously mentioned, the participants were not from a clinical population, and students did not ask to participate because of psychological distresses or conflicts. Thus, consistent with our theoretical foundations, the perceived increase of their metacognitive skills and agency has to be interpreted within Bion’s (1962) concept of *learning from experience* rather than as a therapeutic outcome. Final-year undergraduate students, indeed, are leaving the reassuring university institution that has housed them for at least five years and whose function has been to act as a holding environment (Winnicott, 1965) promoting the acquisition of new competencies to apply to future work scenarios and activating awareness and change processes (Amodeo et al., 2017). This particular period might activate a potential developmental crisis, representing an important turning point in the life of a young adult. The laboratory described above can therefore represent a sort of ‘buffer’ able to alleviate the separation anxiety and to promote a reflective process on one’s own psychological and clinical skills and competencies.

Looking specifically at the results, regarding the perceived improvement of ability of recognizing one’s own emotional states, during the experience participants had the chance to give voice to their still un-thought emotional states. For example, during the second session of a group, a participant stated, “I was finally able to give a name to a physical sensation which I felt in my stomach. I’m angry because I feel that my university path is finishing and I feel alone!” This example shows the capacity of participants to transform physical sensations into thought emotions, thus having the possibility to think and reflect about their unknown emotional states. We believe that this ability might be viewed as an insight about an emotional state, and through Bion’s (1962) lens, as a transformation of beta elements into alpha elements, that is, the transformation of sensory impressions into thoughts that can be dreamed.

On the other hand, we found that participants felt more able to understand causal relationships between their own behaviors and goals to reach. It seems possible to us to track this finding through the ability of participants to reflexively think about their actual student status as connected with their future and imminent status as psychologists. For instance, in the last session of a group, a participant stated:

A retrospective eye is ever necessary… an eye on what one is and on what one was, on what one would like to be… it is necessary to think about oneself as a liquid magma that becomes crystal and then returns to be malleable, in a continuous mutation…

The image of the liquid magma that goes through a structural change was a very powerful stimulus for the group, and the fact that this image was recalled at the end of the experience provided the opportunity to work also on the separation—the group was “liquefying” to find a new shape—and on the new skills acquired. Participants, indeed, expressed the awareness of being in a “liquid” stage of their life, during which they were open to learning from experience and to include into their “magmatic” and still under-construction professional identity new tools and instruments. Moreover, participants were all young adults, and thus they were trying to construct a new lifestyle closer to their current goals and new abilities (Arnett, 2007), because this was a very liquid stage of their lives.

Again, within metacognitive dimensions, participants felt to increase their ability to inferring one’s own and others’ mental states. For instance, during the third session of a group, participants decided to play a game they called “the mirror game.” Some participants answered the question “If I look in the mirror, what am I looking at?”

Following, we report a narrative example by a participant:

I look at a boy who rejects everybody, destroying his most important relationships, an often depressed boy… a boy who does not finish anything, who has also thought to leave university. Nevertheless, when a mirror returns a good image I feel that I become able to do everything I want…as in this case…

This example shows the importance of being mirrored by the group (Esposito et al., 2017b), as this process sets reflexivity in motion, orienting oneself positively towards the future. After the intervention of this participant, indeed, the group started a very fruitful discussion about the skills a psychologist should have to productively do this job. For instance, participants discussed the importance of working through distressed moments and learning to use them to grow up and to perceive themselves as human beings and not only as psychologists. A participant said:

As a future psychologist I believe and hope to be able to think my emotions and sensations, not leaving them to overwhelm me, but using them as a resource both for me and for the other within the clinical relationship.

In this last example, we also believe that the hope of becoming a competent clinical psychologist is comprised. Results suggested that group psychodynamic counselling helped participants to feel more able to derive pathways to desired goals and to motivate themselves via agency thinking to use those pathways. Furthermore, it seems to us that this last narration contains a reference to an agentive position that leads to assume the responsibilities of one’s own actions, thoughts, and emotions. To this end, during the penultimate session of a group, a participant was able to tell his personal story of a past suicide attempt and his long personal therapeutic path before deciding to be enrolled in a clinical psychology university course. The emotional climate of the group in that moment was very warm, and the whole group was able to use that painful experience to reflect on the importance of future clinical psychologists being able to use those experiences to understand and work with the pain of their potential clients. In the meantime, the group had the chance to work through the possibility that psychologists may decide to do this specific job to come to terms with their own pain and internal conflicts. The group was able to activate a reflective process on this complex issue, deeply understanding the importance of reflecting on this need to avoid dangerous relational collusions with potential clients.

This perceived enhancement in the ability of thinking critically about one’s own subjective experience and choices seems to us a fundamental goal that psychologists should achieve, as it allows to use past personal experiences at the service of the actuality. To this end, Kullasepp (2006) stated that the construction of the professional identity for psychologists involves a parallel process that should lead to becoming more and more aware of oneself, of one’s own emotions, cognitions, and so on. It seems to us that participants of our laboratory had the chance to broaden their vision of reality, perceiving it as a complex dimension in which intrapsychic and interpsychic phenomena are constantly intertwined, which is in line with the process highlighted by Kullasepp (2006). In this sense, a participant stated:

This lab has shown me that the relationship I create with others is primarily a sort of dialogue between various instances of my mind, with fantasies, dreams, symbols, representations, models, beliefs… and, never like in this lab, I felt that what I decided to take with me in the bag of my trip… light but well-stocked luggage… is the awareness that we are all connected, that no man is an island, as Pirandello would say.

This last reference to the lab as a trip might be read as a newly achieved awareness that the construction of a professional identity is ever a slow and complex process, during which the subjective experience is strongly intertwined with others, such as colleagues, clients, and supervisors. More generally, we believe that this experiential course enabled students to grow up both subjectively and professionally, becoming one of the tools that students might use in their future careers. To this end, during the last session of a group, a pregnant participant told the group a very distressing dream: she dreamed about killing her daughter and woke up in anxiety. The counsellor had the chance to invite the group to reflect and work through the separation anxiety manifested through the murder of a baby girl. Indeed, the group was turning toward the end and the participants needed to “kill” the group to make room for subjectivity again. Beyond this possible interpretation of the dream and leaving its personal meaning in the background, the counsellor brought participants to reflect on their developmental stage as a phase where they are “killing” their student status—represented by the baby girl—to make room for a new adult status, or rather the imminent status of a clinical psychologist. Thus, through this dream, we believe that participants unconsciously represented the first steps of their individuation process as future professionals.

### Limitations

This study is not free from limitations. First, the sample is not representative of the general population of final-year undergraduates in clinical psychology, first of all because of its quite small number of participants. However, the experiential nature of the intervention allows to overcome this limit. Second, we did not compare our results with a control group of students in clinical psychology not participating in the same experience, which could have given higher reliability to the results obtained. In addition, comparing the results obtained with those from a control group may have helped in understanding whether other variables might intervene in the enhancement of students’ metacognitive skills and agency, such as the graduation process itself. Thus, future research should consider involving a control group to ensure that the perceived change in metacognitive skills and agency levels are due to the specific experience, excluding the influence of other possible variables. However, although this is an important limit of the study making difficult to assess causality, at the same time it should be considered as partial due to the three-month follow-up assessment that demonstrated an enduring increase in these dimensions also after the immediate end of the experience. Finally, the measures used in this study were subjective self-evaluations based on self-report. As such, the results may be better understood as reflecting the participants’ confidence in each of these domains rather than their ability per se. Future research should considering to assess metacognitive and agentive skills through non-self-report measures evaluating objective improvements rather than self-perceptions.

### Conclusions and Future Directions

Educational institutions should ensure practical experiences where students might be able to learn from experience, adding new tools and skills to their repertoire. Providing experiential spaces aimed at enhancing metacognitive skills and agency through specific attention to the emotional dimension helps to increase reflective thinking and to promote reflective processes that have the functions of both alleviating anxieties for the future and activating awareness and change processes.

Future research might consider applying this clinical training methodology to students in different degree courses, adapting the laboratory to specific competencies that these students should acquire for their future professions. Moreover, it would be interesting to test the effectiveness of this device for first-year students who have had recent access to the university and to repeat the same experience at the end of their university path, assessing the possible changes that may occur.
